# Antimicrobial efficacy of *Bifidobacterium longum* FB1-4 cell-free supernatant against MRSA: insights into mechanisms and food matrix application

**DOI:** 10.1128/spectrum.00991-25

**Published:** 2026-03-02

**Authors:** Jing Wang, Qisijing Liu, Yarui Liu, Jin Wang, Yu Jiang, Xuemeng Ji, Xinyang Li, Yaxiong Song, Jingmin Liu, Shuo Wang

**Affiliations:** 1 Tianjin Key Laboratory of Food Science and Health, School of Medicine, Nankai University 481107, Tianjin, China; 2 College of Environmental Science and Engineering, Nankai University, Tianjin, China; University of Turin, Gruglisco, Turin, Italy

**Keywords:** *Bifidobacterium longum *FB1-4, MRSA, drug resistance inhibition, antimicrobial activity, food matrix, biofilm

## Abstract

The food safety landscape faces a significant challenge from methicillin-resistant *Staphylococcus aureus* (MRSA), a virulent and antibiotic-resistant foodborne pathogen. This study investigates the antimicrobial properties and mechanisms of cell-free supernatant (CFS) from *Bifidobacterium longum* FB1-4 against MRSA. The CFS exhibited robust antibacterial activity, achieving a minimum inhibitory concentration (MIC) of 5.56 mg/mL and maintaining a bacterial elimination rate of over 80% for 8 h at 2× MIC, demonstrating superior sustained bactericidal efficacy compared to vancomycin. Antibacterial component analysis revealed that while the initial effect is partly pH-dependent, the activity is largely attributable to heat-stable molecules, retaining 90% efficacy after high-temperature treatment. In a food matrix, FB1-4 CFS exhibited impressive bactericidal effects against three MRSA strains, leading to elimination rates of 94.64% at 2 h, 98.02% at 4 h, 99.90% at 6 h, and nearly complete eradication (99.99%) by 8 h. Furthermore, FB1-4 CFS significantly suppressed all three tested MRSA strains (MRSA-43300, MRSA-337371, and MRSA-361194; *P* < 0.05), *mecA* expression, increased antibiotic susceptibility, and diminished biofilm formation by 70%. Transcriptomic and metabolomic analyses revealed that FB1-4 CFS modulates two-component systems and quorum sensing, leading to reduced virulence factor expression. *In vivo* studies confirmed a 90% reduction in MRSA colonization in mouse intestines. These findings provide critical insights into how probiotic metabolites inhibit MRSA, underscoring their potential as natural therapeutic agents in food safety.

**IMPORTANCE:**

This study unveils a novel, multi-targeted strategy against methicillin-resistant *Staphylococcus aureus* (MRSA) using the cell-free supernatant (CFS) of *Bifidobacterium longum* FB1-4. Integrating multi-omics analyses, we systematically demonstrate that FB1-4 CFS acts synergistically to disrupt MRSA’s antibiotic tolerance, biofilm formation, virulence (via the SaeR/S system), and core metabolism. This multifaceted mechanism circumvents the resistance risk of single-target antibiotics. FB1-4 CFS also shows significant efficacy in both food matrices and an *in vivo* model, highlighting its dual potential as a natural food preservative and a therapeutic candidate to combat drug-resistant infections.

## INTRODUCTION

Methicillin-resistant *Staphylococcus aureus* (MRSA) presents a critical challenge to food safety due to its virulence and prevalence in food production environments (1, 2). The challenge of treating MRSA infections is further complicated by the emergence and spread of antimicrobial resistance (AMR), contributing substantially to the global infectious disease burden (2). The World Health Organization estimates that drug-resistant infections could lead to 10 million deaths annually by 2050 (3). Therefore, there is an urgent need to develop innovative antibacterial strategies that effectively target drug-resistant pathogens like MRSA and mitigate the transfer of resistance determinants. These strategies are crucial to combat the growing threat of AMR and safeguard global health.

The horizontal transfer of the well-known resistance gene *mecA* in MRSA represents the primary cause of widespread AMR (4). The *mecA* gene encodes proteins that reduce the affinity of β-lactam antibiotics, leading to decreased drug susceptibility in MRSA and consequently increasing the difficulty of therapeutic interventions (5). The treatment complexity is further exacerbated by the ability of MRSA to form biofilms, which are microbial communities encased in self-produced extracellular matrix (6). These biofilms adhere to surfaces and are protected by extracellular polysaccharides (EPS), providing a protective barrier against host immune responses and antibiotic penetration (7, 8). Consequently, bacteria within biofilms exhibit significantly enhanced AMR compared to their planktonic counterparts, making MRSA infections particularly challenging to eradicate.

Recent investigations have revealed that both the two-component regulatory system SaeR/S and quorum-sensing system play essential roles in MRSA biofilm formation and virulence factor regulation (9, 10). The SaeR/S system regulates the expression of various MRSA virulence factors, including hemolysin A (*hla*), γ-hemolysin (*hlgABC*), and coagulase (*can*) (11, 12). The quorum-sensing system modulates multiple virulence phenotypes, including the secretion of extracellular proteases, production of α-hemolysin, and bacterial motility (13, 14). Based on these findings, the SaeR/S regulatory system and quorum-sensing system represent potential therapeutic targets for inhibiting MRSA virulence.

Probiotics, as natural inhabitants of the human gut with recognized health benefits, have garnered increasing interest as a potential source of novel antimicrobials (15, 16). Their diverse physiological activities, including antibacterial, antioxidant, and immunomodulatory effects, stem from a repertoire of bioactive compounds, including organic acids, short-chain fatty acids, and antimicrobial peptides (17–19). While research has predominantly focused on the antimicrobial potential of lactic acid bacteria and their metabolites, the potential of *Bifidobacterium longum* (*B. longum*) against MRSA remains underexplored.

This provides novel insights, first revealed by the inhibitory activity of *B. longum* metabolites against MRSA and their potential applications in food matrices. Through comprehensive characterization of the antibacterial and anti-biofilm efficacy of FB1-4 cell-free supernatant (CFS), coupled with transcriptomic and metabolomic analyses, the research systematically investigated the compound’s impact on two-component regulatory systems and quorum-sensing mechanisms that modulate MRSA virulence factors. The study elucidated the underlying molecular targets and pathways, providing novel insights into the development of food-grade preservatives against drug-resistant bacteria. These findings establish a significant foundation for innovative strategies in combating AMR through natural microbial metabolites.

## MATERIALS AND METHODS

### Biological materials and reagents

Three MRSA strains, namely ATCC43300 (MRSA-43300), BNCC 337371 (MRSA-337371), and BNCC361194 (MRSA-361194), were obtained from Beijing Northern Biotechnology Co., Ltd. (Beijing, China). The experimental strain FB1-4, identified as *B. longum subsp. longum*, was obtained from Tianjin Key Laboratory of Food Science and Health (School of Medicine, Nankai University).

The animal study employed 16 male BALB/c mice (age: 8 weeks) maintained under standardized conditions (22°C ± 2°C, 40%–60% relative humidity) with alternating 12-h light-dark cycles. Standard feed and portable water were accessible to all animals during the study period. All experimental protocols were conducted under ethics committee authorization (approval code: 2024-SYDWLL-000701). All experimental procedures adhered to relevant ethical guidelines to ensure the welfare of the animals.

Oxoid (Basingstoke, Hampshire, UK) provided MRS broth, while L-cysteine was sourced from Maokang Biotechnology (Shanghai, China). Mupirocin was purchased from MedChemExpress (Monmouth Junction, NJ, USA). Sterile horse serum was acquired from HyClone (GE Healthcare Life Sciences, Logan, UT, USA). Luria-Bertani (LB) medium and agar were obtained from Oxoid (Thermo Fisher Scientific, Basingstoke, UK).

### Isolation and purification of probiotics

Breast milk and infant fecal samples were serially diluted and plated on solid selective media. After aerobic or anaerobic incubation at 37°C for 24–48 h, colonies resembling *Bifidobacterium* or *Lactobacillus* were repeatedly streaked for purification. Purified isolates were cultured in liquid MRSC or MRS broth for further identification. The *Bifidobacterium*-selective medium consisted of MRS base supplemented with filter-sterilized horse serum (50 mL/L), 0.05% L-cysteine hydrochloride, and 0.05 mg/mL mupirocin (added after autoclaving). MRSC liquid medium was MRS broth containing 0.05% L-cysteine hydrochloride.

### Antimicrobial activity assay

MRSA was cultured overnight in LB broth at 37°C and 200 rpm until it reached the logarithmic growth phase (unless otherwise specified, the MRSA culturing protocol described in section Antimicrobial Activity Assay was consistently applied throughout the study). FB1-4 was cultured in MRS broth under anaerobic conditions at 37°C. Following centrifugation at 8,000 × *g* for 5 min, the supernatant was collected and filtered through a 0.2 μm sterile filter to obtain FB1-4 CFS. Using the Oxford cup method (agar diffusion method using stainless steel cylinders [20]), the antibacterial activity of FB1-4 CFS against MRSA was evaluated. Briefly, the optical density at 600 nm (OD_600_) of the overnight MRSA culture was adjusted to 0.1–0.2 and spread evenly onto LB agar plates. Sterile Oxford cups were placed on the agar surface, with one cup positioned in the center serving as a negative control. Subsequently, 200 μL of FB1-4 CFS, a volume sufficient to form a confluent inhibition zone, was added to each test cup, while the control cup received an equal volume of sterile MRS broth. Incubation of plates occurred at 37°C for 12–18 h. A vernier caliper was used to measure the diameter of the inhibition zone around each cup. All experiments were conducted in triplicate.

#### Minimum inhibitory concentration determination

The minimum inhibitory concentration (MIC) of FB1-4 CFS against MRSA was determined following Clinical and Laboratory Standards Institute guidelines (21). MRSA strains were grown overnight to logarithmic phase, standardized to 0.5 McFarland, and diluted to 5 × 10⁵ CFU/mL in LB broth. FB1-4 CFS underwent serial twofold dilutions. Equal volumes of diluted FB1-4 CFS and MRSA suspension (200 μL total) were incubated in 96-well plates at 37°C for 18–24 h. Controls included LB broth and undiluted FB1-4 CFS. The MIC was determined as the lowest concentration inhibiting 90% of bacterial growth (MIC 90), measured by OD_600_ using a microplate reader. Experiments were performed in triplicate.

Finally, a volume of 20 mL of FB1-4 CFS was subjected to lyophilization to convert the MIC unit from a volume-by-volume (vol/v) concentration to a mass-to-volume (mg/mL) concentration (22).

#### Growth curves

In a 96-well microplate, FB1-4 CFS was serially diluted twofold with LB broth to achieve concentrations ranging from 0 to 4× MIC (100 μL per well). An equal volume of diluted MRSA suspension was added to each well. Optical density at 600 nm was monitored every 120 min using a SpectraMax iD3 microplate reader (Molecular Devices, San Jose, CA, USA).

#### Vancomycin control

To compare the antibacterial effects of FB1-4 CFS with commonly used antibiotics for MRSA treatment, vancomycin was selected as a positive control. The bactericidal activity of FB1-4 CFS and vancomycin against MRSA was assessed over time. As previously described, the MIC of vancomycin against MRSA was determined. The MRSA culture in the logarithmic growth phase was adjusted to an OD_600_ of 0.2 and mixed in a 1:1 ratio with vancomycin at both MIC and 2× MIC concentrations, respectively, along with FB1-4 CFS. Samples were taken at 2, 4, 6, and 8 h post-treatment, diluted, and plated to determine the viable bacterial count. The relative survival rate was calculated based on the viable count of the control group (untreated).

#### Elimination rate of MRSA by FB1-4 CFS in food matrix

Three MRSA strains were individually cultured overnight to mid-logarithmic phase and standardized to 10^6^ CFU/mL. Under aseptic conditions, pre-manufactured sausage products were sectioned into 5 g square segments and surface-decontaminated using UV irradiation for 15 min.

Subsequently, 100 μL of standardized bacterial suspension was precisely pipetted onto the sample surface and allowed to adsorb for 15 min. FB1-4 CFS was diluted to 2× MIC, accounting for potential matrix absorption limitations. Two milliliters of diluted FB1-4 CFS was uniformly dispensed onto the sample surface and permitted to absorb completely. Control samples received sterile physiological saline. Inoculated samples were incubated at 37°C, with sampling conducted at 2-, 4-, 6-, and 8-h intervals. For microbial enumeration, each sample was homogenized in 40 mL sterile physiological saline for 2 min, followed by decimal dilution and spread-plating. Plates were incubated overnight at 37°C, after which bacterial colonies were quantitatively assessed.

### Effects of FB1-4 CFS on MRSA antibiotic resistance

FB1-4 CFS (at MIC) was added to triplicate cultures of diluted MRSA suspensions (10⁶ CFU/mL). In parallel, a control group without FB1-4 CFS was prepared. Both groups were incubated at 37°C with 200 rpm shaking for 12–18 h. RNA was extracted using the PureLink RNA Mini Kit (Thermo Fisher Scientific) with a *Staphylococcus*-specific lysis buffer. The total RNA was extracted from both groups and reverse-transcribed to cDNA. The expression of the antibiotic resistance gene *mecA* was analyzed using reverse transcription quantitative polymerase chain reaction (RT-qPCR). To evaluate the effects of FB1-4 CFS on gene expression, reactions were conducted using SYBR Green PCR Master Mix on a QuantStudio 3 Real-Time PCR System (both from Thermo Fisher Scientific, Waltham, MA, USA). Table 1 lists the primer sequences used for RT-qPCR.

**TABLE 1:** Primers used in this study

Primers	Sequence (5′−3′)
*16S rRNA*_F	CCATAAAGTTGTTCTCAG TT
*16S rRNA*_R	CATGTCGATCTACGATTACT
*mecA*_F	TGGAACTTGTTGAGCAGAGGT
*mecA*_R	TGGAACTTGTTGAGCAGAGGT
*psmα*_F	ACCCATGTGAAAGACCTCCTTTGT
*psmα*_R	ATGGGTATCATCGCTGGCATC
*Ica*_F	TCGCACTCTTTATTGATAGTCGCTACGAG
*Ica* _R	TGCGACAAGAACTACTGCTGCGTTAAT
*hlgA_*F	GATGCCCTAGTTGTTAAGATG
*hlgA_*R	TTTCCGCCGATATTATAGCC
*hlgC_*F	GAATCTACAAACGTGAGTCA
*hlgC_*R	TTTGACCTGATTCAGTGGC
*saeR_*F	GTCGTAACCATTAACTTCTG
*saeR_*R	ATCGTGGATGATGAACAA
*saeS_*F	TGTATTTAAAGTGATAATATGAGTC
*saeS_*R	CTTAGCCCATGATTTAAAAACACC

The experimental design aimed to investigate the effect of FB1-4 CFS on the drug sensitivity of MRSA, involving four distinct groups. Control group A was treated with the MIC of methicillin only, while experimental group B received 1/4 MIC of FB1-4 CFS (a concentration that does not affect normal MRSA growth) alongside the MIC of methicillin. Experimental group C was treated with 1/2× MIC of FB1-4 CFS (also non-inhibitory) in combination with the MIC of methicillin, and control group D had no added solution. In each group, 100 μL of the respective solution was mixed with 100 μL of MRSA logarithmic growth phase culture, adjusted to an OD_600_ of 0.2, and co-cultured in a 96-well plate at 37°C for 16–18 h. The OD_600_ absorbance values of all groups were measured using a microplate reader to assess bacterial growth.

### Biofilm inhibition assay

MRSA suspensions were inoculated into LB medium with and without sub-inhibitory concentrations of FB1-4 CFS. Following cultivation, total bacterial RNA was extracted and reverse-transcribed to cDNA. Expression changes in biofilm-related genes (*Psma*, *Ica*) were quantified using qPCR, with 16S rRNA as the internal reference gene. Fold changes in gene expression were calculated using the 2^−ΔΔCt^ method. Each experiment was performed in triplicate, and significant differences between treatment and control groups were evaluated using a t-test (*P* < 0.05).

As described by Ferrières et al. (23), biofilm formation was quantified using a crystal violet staining method with minor modifications. MRSA cultures were treated with vancomycin and FB1-4 CFS at the 2× MIC and 4× MIC concentrations to assess biofilm formation. The biofilm quantification assay was then performed following the protocol outlined by Ferrières et al. (23), with absorbance measured at 590 nm using a microplate reader.

The biofilm formation assay was performed as described by Shao et al. (24) with slight modifications. Briefly, MRSA cultures were treated with FB1-4 CFS at final concentrations of 0, 2× MIC, and 4× MIC. Samples were critically point dried using a Tousimis Autosamdri-815 Series dryer and sputter-coated with gold using a HITACHI MC1000 coater. A Hitachi SU8020 field emission scanning electron microscope (SEM; Hitachi High-Technologies, Tokyo, Japan) equipped with a HORIBA EMAX mics2 energy dispersive spectrometer was used for examination. Images were captured at 5 kV accelerating voltage with a 50 nm point resolution.

The effects of FB1-4 CFS on EPS production in MRSA were evaluated using a modified phenol-sulfuric acid method (25). The MRSA isolates were incubated in LB medium containing FB1-4 CFS at concentrations of 0, 1/4× MIC, 1/2× MIC, 2× MIC, and 4× MIC. To evaluate the bactericidal efficacy relative to vancomycin, control groups were established by supplementing the medium with equivalent concentrations of vancomycin. Following incubation at 37°C for 18 h, bacterial cells were harvested by centrifugation (8,000 × *g*, 10 min), and the pellets were resuspended in phosphate-buffered saline. The suspension was centrifuged again under identical conditions. EPS was precipitated by mixing the resulting supernatant with an equal volume of absolute ethanol. A mixture was prepared by adding 1 mL of EPS solution to 1 mL of chilled 5% phenol, followed by the introduction of 5 mL 98% sulfuric acid. The resulting solution’s optical density was quantified at wavelength 490 nm via spectrophotometry. The percentage reduction in EPS content was calculated based on the absorbance values.

### Analysis of antimicrobial components

#### Effect of pH on antimicrobial activity

To determine whether the antibacterial activity of FB1-4 CFS is primarily due to acidic metabolites, the pH of MRS medium was adjusted to match that of FB1-4 CFS using phosphate buffer. An MRSA suspension (OD₆₀₀ = 0.2) was inoculated into the following groups: (i) FB1-4 CFS at MIC concentration, (ii) pH-adjusted MRS medium (acidic control), and (iii) LB medium (normal growth control). All groups were incubated at 37°C, and the absorbance at 600 nm was measured every 2 h using a microplate reader. The inhibition rates of MRSA in the FB1-4 CFS and pH-adjusted MRS groups were calculated accordingly. Each experiment was performed in triplicate.

#### Effect of heat treatment on antimicrobial components

To evaluate the thermal stability of the active constituents in FB1-4 CFS, the supernatant was adjusted to the following concentrations: crude (1× MIC), 2× MIC, 1× MIC, and 1/2× MIC. Each concentration was heat-inactivated by autoclaving at 121°C for 15 min, with untreated CFS serving as the control. MRSA suspension (OD₆₀₀ = 0.2) was inoculated into untreated FB1-4 CFS, heat-inactivated FB1-4 CFS, and LB medium (control), followed by static culture at 37°C for 12 h. The absorbance at 600 nm was measured, and the inhibition rate was calculated. All assays were independently repeated three times.

### Genome sequencing, assembly, and annotation

Draft genome assemblies were constructed using short-read sequences generated by next-generation sequencing. Reads were assembled *de novo* using SOAPdenovo2 with multiple k-mer values to optimize contig assembly. Complete genome sequences were generated using long-read sequencing data assembled with Unicycler, followed by error correction with Pilon. Circular chromosomes and plasmids were identified based on overlapping sequences at the termini. Plasmids were identified using PlasFlow and annotated using BLAST against the PLSDB database (https://ccb-microbe.cs.uni-saarland.de/plsdb/).

Protein-coding sequences (CDSs) were predicted from assembled genomes using Glimmer, GeneMarkS, or Prodigal, depending on the genome completeness and sequence type. Using tRNAscan-SE v2.0 and Barrnap, transfer RNAs (tRNAs) and ribosomal RNAs (rRNAs) were identified, respectively. Predicted CDSs were functionally annotated based on homology to sequences in the Gene Ontology (GO) database. The genome circle map for *B. longum* FB1-4 was generated using the CGView web server (https://github.com/paulstothard/cgview).

### Transcriptome analysis

MRSA isolates were cultured in the presence or absence of FB1-4 CFS at the MIC. RNA extraction and quality assessment were conducted as previously described (26). RNA-seq libraries were prepared and sequenced following established protocols (27).

Differential gene expression analysis employed DESeq2 (version 1.20.0) for samples with biological replicates, using a negative binomial model and Benjamini-Hochberg adjusted *P*-values (<0.05 for significance) (28). For samples lacking biological replicates, edgeR (version 3.22.5) normalized read counts and performed differential expression analysis, with *q*-values < 0.005 and |log2(fold change) | > 1 considered significant. GOseq performed GO enrichment analysis, correcting for gene length bias (*P* <0.05 significant). Kyoto Encyclopedia of Genes and Genomes (KEGG) pathway enrichment analysis utilized the KEGG database (https://www.kegg.jp/kegg/pathway.html) and KOBAS software (29–32).

### Effect of FB1-4 CFS on expression of key metabolic genes

Based on previous transcriptome analysis, key genes were selected for expression analysis. MRSA suspensions were cultured in LB medium with and without sub-inhibitory concentrations of FB1-4 CFS. Total bacterial RNA was extracted and reverse-transcribed to cDNA. Target gene expression levels were quantified using qPCR as described above, with 16S rRNA as the internal reference gene. Fold changes in gene expression were calculated using the 2^−ΔΔCt^ method. All experiments were performed in triplicate.

### Non-targeted metabolomics analysis protocol

We isolated MRSA cultures as described in section Genome Sequencing, Assembly, and Annotation. The method included lyophilizing 1 mL of the sample, then resuspending it in pre-chilled 80% methanol and vigorously vortexing. After a 5-min incubation on ice, samples were centrifuged at 15,000 × *g* for 15 min at 4°C. A portion of the supernatant was diluted with LC-MS grade water to achieve a final methanol concentration of 53%. This dilution was transferred to fresh Eppendorf tubes and subjected to another centrifugation under the same conditions. The resulting supernatant was collected for LC-MS/MS analysis.

Metabolomic profiling utilized a Vanquish UHPLC system (ThermoFisher, Germany) linked with an Orbitrap Q Exactive HF or HF-X mass spectrometer (ThermoFisher, Germany). Separation was performed on a Hypersil Gold column (100 × 2.1 mm, 1.9 μm) at a flow rate of 0.2 mL/min over a 12-min linear gradient. The mobile phases were 0.1% formic acid in water (A) and methanol (B) for both positive and negative ionization. The gradient profile was set to 2% B (0–1.5 min), 2%–85% B (1.5–3 min), 85%–100% B (3–10 min), 100%–2% B (10–10.1 min), and back to 2% B (10.1–12 min). The parameters for the Q Exactive HF included a spray voltage of 3.5 kV, capillary temperature at 320°C, sheath gas flow of 35 psi, auxiliary gas flow at 10 L/min, S-lens RF level of 60, and auxiliary gas heater at 350°C.

Raw data were processed using Compound Discoverer 3.3 software for peak alignment, extraction, and quantification of metabolites. Identification involved spectral matching with mzCloud, mzVault, and MassList databases, followed by KEGG annotation. Quality control excluded compounds with CV values exceeding 30% in QC samples, normalizing peak intensities to total spectral intensity. Differential metabolites were classified with VIP scores >1, *P*-values <0.05, and fold changes ≥2 or ≤0.5. Multivariate analyses included PCA and PLS-DA with metaX software, while hierarchical clustering analysis was performed on z-score normalized metabolite intensities. Pathway enrichment analysis considered pathways significant when the ratio of differential to total identified metabolites exceeded the background ratio, with a *P*-value <0.05.

### Effect of FB1-4 CFS on virulence factors regulated by quorum sensing

This study evaluated the impact of sub-MIC levels of FB1-4 CFS on MRSA protease secretion. The bacterial strains underwent cultivation at 37°C for 24 h in LB medium fortified with FB1-4 CFS at 1/2× MIC concentration. The resulting supernatant was obtained via centrifugation and sterilized through a 0.22 μm pore-size filter. Then, 100 μL of the sterile supernatant was transferred into wells on skim milk agar plates (1.5% LB agar plus 5% skim milk). After a 24-h incubation at 37°C, protease activity was gauged by measuring the diameters of the clear zones. Experiments were carried out in triplicate (33).

To investigate how FB1-4 CFS affects MRSA hemolysin production, an MRSA cell suspension (approximately 10⁸ CFU/mL) was inoculated at 1% (vol/vol) into LB medium supplemented with sub-inhibitory FB1-4 CFS (0, 1/8, 1/4, and 1/2× MIC) and incubated for 24 h at 37°C with shaking. The supernatant was harvested by centrifugation (10,000 rpm, 4°C, 5 min) and further filtered through a 0.22 μm membrane filter. A 475 μL portion of the filtered supernatant was combined with 25 μL of defibrinated rabbit blood and incubated for 2 h at 37°C. Post-centrifugation (2 min at 5,000 rpm), optical density measurements were performed at 540 nm using a microplate spectrophotometer. The hemolysis inhibition rate was obtained through triplicate assays, with sterile LB medium utilized as the baseline control (34).

Motility assessment was employed on LB medium containing 0.3% (wt/vol) agar. After autoclaving (121°C, 15 min) and cooling to approximately 50°C, sub-inhibitory concentrations of FB1-4 CFS were introduced. Each Petri plate was poured with 20 mL of this medium to maintain uniform thickness, set at room temperature for 30 min to solidify, and then dried at 37°C for 1 h to eliminate surface moisture. MRSA strains were cultured overnight in 5 mL LB broth at 37°C, and the bacterial suspension was adjusted to OD_600_ = 0.1–0.2 using fresh LB broth. Two microliters of the standardized suspension was carefully placed at the center of each plate (ensuring no contact between pipette tip and agar), after which the plates were inverted and incubated for 24 h at 37°C. MRSA motility was documented, with each experiment performed in triplicate (35).

### *In vivo* mice experiment

Sixteen 8-week-old male BALB/c mice were randomly assigned to two groups (*n* = 8/group). An *in vivo* MRSA ATCC 43300 infection model was established by oral gavage with 1.0 × 10^8^ CFU of bacterial suspension. Six hours post-infection, the control group received sterile saline via oral gavage, while the treatment group received 200 μL of FB1-4 CFS. Mice were monitored daily for weight changes over a 4-day observation period. On day 4, colon tissue samples (the primary site of MRSA colonization) were collected to determine bacterial load (CFU) in both groups. Blood samples were also collected for serum TNF-α quantification.

### Statistical analysis

For statistical evaluations, we utilized GraphPad’s data analysis tool, Prism 9 (GraphPad Software, La Jolla, CA, USA). Differences between groups were evaluated by one-way ANOVA with Dunnett’s post hoc test. Results were not considered statistically insignificant unless *P* ≥ 0.05.

## RESULTS

### Antimicrobial activity

#### Bactericidal efficacy of FB1-4 CFS against MRSA and comparative analysis with vancomycin

**Fig 1:**
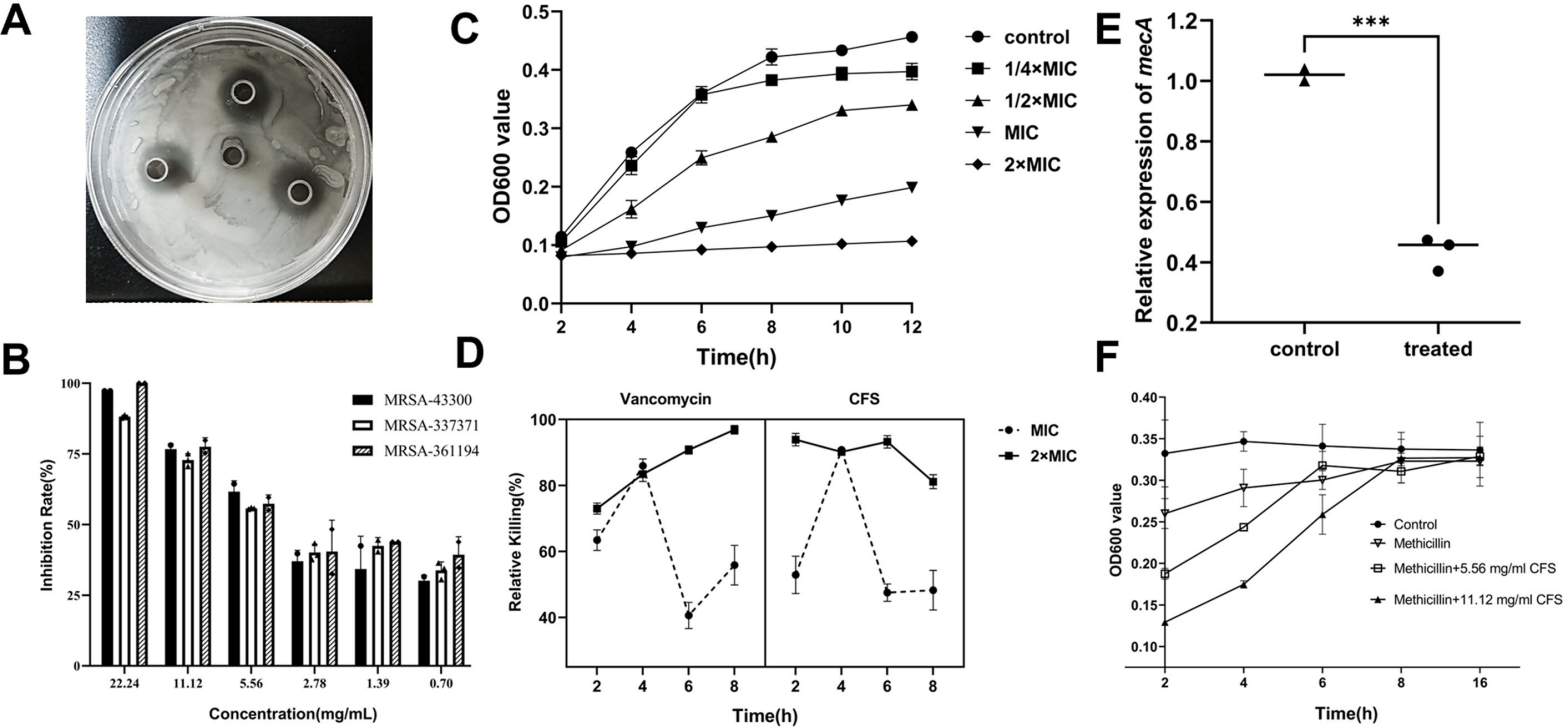
Antimicrobial activity of FB1-4 CFS against MRSA. (**A**) Oxford cup assay showing zones of inhibition. (**B**) MIC determination of FB1-4 CFS against MRSA, with OD values measured at different concentrations. (**C**) Growth curves of MRSA at various sub-MIC and supra-MIC concentrations of FB1-4 CFS. (**D**) Time-kill assay comparing the bactericidal effects of FB1-4 CFS and vancomycin against MRSA at MIC and 2× MIC concentrations. Error bars represent standard deviation from triplicate experiments. (**E**) Relative expression of *mecA* in MRSA following treatment with FB1-4 CFS. (***P < 0.001). (**F**) Growth curves of MRSA under different treatment conditions. Control: untreated MRSA; Methicillin: MRSA treated with methicillin alone; Methicillin + 5.56 mg/mL CFS and Methicillin + 11.12 mg/mL CFS: MRSA treated with methicillin and 5.56 mg/mL or 11.12 mg/mL FB1-4 CFS, respectively. Error bars represent standard deviation from triplicate experiments.

The antimicrobial efficacy of the FB1-4 CFS against MRSA was assessed through an agar well diffusion assay (Fig. 1A). The average inhibition zone diameters for FB1-4 CFS against MRSA-43300, MRSA-337371, and MRSA-361194 were 18.20, 18.10, and 17.70 mm, respectively. Broth microdilution results indicated that 5.56 mg/mL FB1-4 CFS inhibited 90% bacterial growth, establishing this concentration as the MIC90 (Fig. 1B). Growth of MRSA was significantly inhibited at the MIC within 8 h of treatment. At 2× MIC, the OD_600_ remained relatively stable between 0.1 and 0.2 throughout the 12-h incubation period, indicating substantial inhibition of bacterial growth (Fig. 1C).

Comparative experimental results of FB1-4 CFS and vancomycin demonstrated that at 2× MIC concentration, vancomycin exhibited a potent and sustained bactericidal effect, with a killing rate approaching 100% after 8 h. However, at the MIC concentration, the bactericidal activity of vancomycin was significantly reduced, reaching a peak killing rate of approximately 85% in 4 h, and then rapidly declining to 55% at 8 h, indicating that its bactericidal activity is concentration-dependent, and its sustained killing capability is insufficient at lower concentrations. In contrast, the FB1-4 CFS maintained a killing rate above 80% within 8 h at 2× MIC concentration, demonstrating a more stable bactericidal effect compared to vancomycin. Although at the MIC concentration, the killing rate of FB1-4 CFS peaked at around 90% in 4 h and then declined, it ultimately reached approximately 50%, still outperforming the performance of vancomycin at the same concentration (Fig. 1D). These results suggest that FB1-4 CFS has significant bactericidal activity against MRSA and exhibits more persistent antimicrobial effects at higher concentrations.

#### Antimicrobial efficacy evaluation of FB1-4 CFS against MRSA in food matrix

The antibacterial potential of FB1-4 CFS was systematically investigated against three MRSA strains over an 8-h experimental period. An immediate and robust bactericidal effect was observed, with an initial elimination rate of 94.64% at 2 h, which progressively intensified to 98.02% by 4 h. The antimicrobial efficacy continued to escalate, reaching 99.90% at 6 h and approaching near-complete bacterial eradication (99.99%) by 8 h.

While control samples demonstrated unrestrained bacterial proliferation, increasing from 8.4 × 10^4^ to 1.28 × 10^7^ CFU/g, treated samples exhibited a dramatic reduction in bacterial load from 4.5 × 10^3^ to 1.0 × 10^3^ CFU/g (Table 2). These findings conclusively demonstrate the potent and sustained antibacterial activity of FB1-4 CFS against MRSA within the food matrix.

**TABLE 2:** Time-dependent bactericidal efficacy of FB1-4 CFS against MRSA in food matrix^*a*^

Time (h)	Control group (CFU/g)	Treatment group (CFU/g)	Elimination rate (%)
2	8.4 × 10⁴	4.5 × 10³	94.64
4	2.5 × 10^6^	4.95 × 10⁴	98.02
6	5.22 × 10^6^	5.0 × 10³	99.05
8	1.28 × 10^7^	1.0 × 10³	99.92

^*a*^ Quantitative assessment of MRSA population dynamics in a simulated food environment over an 8-h intervention period. Experimental groups included a baseline culture supplemented with sterile saline, contrasted with cultures exposed to FB1-4 CFS. Bacterial population reduction was determined through comparative logarithmic analysis between control and treatment conditions.

#### Inhibition of gene expression and effects on drug sensitivity of MRSA

The experimental results of FB1-4 CFS on the inhibition of the MRSA resistance gene *mecA* and antibiotic susceptibility testing showed that, compared to untreated controls, expression of the antibiotic resistance gene *mecA* decreased by 58.7% (*P* < 0.001, Fig. 1E). The experimental results shown in Fig. 1F indicate that compared to the control group A, the OD_600_ values of the experimental groups B and C with added FB1-4 CFS were significantly reduced, and the degree of reduction was positively correlated with the FB1-4 CFS concentration. Control group D showed the highest OD_600_ value, reflecting the normal growth of MRSA without inhibition. These results suggest that the addition of FB1-4 CFS can enhance the sensitivity of MRSA to methicillin, and this enhancing effect is dose-dependent. In summary, FB1-4 CFS can reduce the expression of the methicillin resistance gene *mecA* and increase the drug sensitivity of MRSA to methicillin.

### Inhibition of biofilm formation

**Fig 2:**
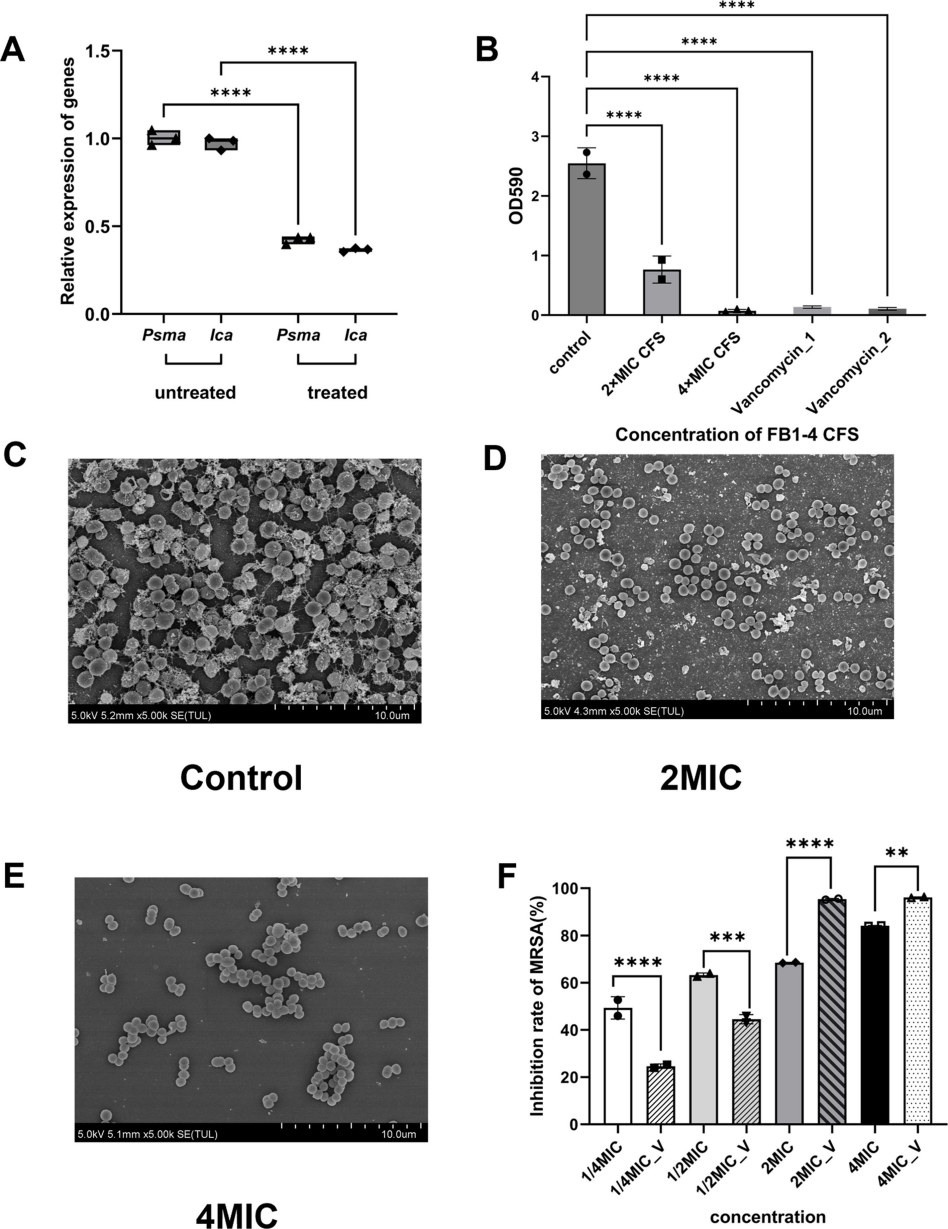
Effects of FB1-4 CFS on MRSA biofilm formation and associated gene expression. (**A**) Relative expression of biofilm-related genes (*Psma* and *Ica*) in MRSA before and after FB1-4 CFS treatment, as determined by RT-qPCR. (**B**) Quantitative analysis of biofilm formation measured by optical density (OD_590_) following treatment with different concentrations of FB1-4 CFS and vancomycin. Control represents untreated MRSA. Data are presented as mean ± SD (*n* = 3). (**C–E**) SEM images showing MRSA biofilm formation under different conditions: (**C**) untreated control, (**D**) 2MIC FB1-4 CFS treatment, and (**E**) 4MIC FB1-4 CFS treatment. Scale bars: 10 μm. (**F**) Quantification of EPS production at different concentrations of FB1-4 CFS and vancomycin, measured by OD490. Data are presented as mean ± SD (*n* = 3). Statistical significance is indicated by (*P* < 0.0001) compared to the control. **P < 0.01；***P < 0.001；****P < 0.0001.

This study investigated the impact of FB1-4 CFS on MRSA biofilm formation (Fig. 2A through F). qPCR analysis revealed that sub-inhibitory concentrations of FB1-4 CFS reduced expression of biofilm-related genes *Psma* and *Ica* by 62.4% and 60.7%, respectively (Fig. 2A).

Biofilm formation assays showed control group OD_590_ values of 2.5–2.8, indicating robust bacterial growth. Treatment with FB1-4 CFS at the 2× MIC resulted in approximately 70% biofilm inhibition (*P* < 0.0001). At the 4× MIC, OD values decreased to near zero, indicating near-complete growth inhibition (*P* < 0.0001), demonstrating a concentration-dependent inhibitory effect. Vancomycin, used as a positive control, also showed near-complete inhibition at both 2× MIC and 4× MIC concentrations (*P* < 0.0001). While FB1-4 CFS exhibited less potent inhibition than vancomycin at the 2× MIC, it demonstrated comparable efficacy at 4× MIC (Fig. 2B). SEM further revealed the impact of FB1-4 CFS on biofilm architecture. Untreated MRSA biofilms displayed tightly packed cells embedded in a dense extracellular matrix. Treatment with 2× MIC FB1-4 CFS resulted in increased surface cracks, loosening of the extracellular matrix, and disrupted biofilm structure. At 4× MIC, significant biofilm damage was observed, characterized by large fissures, loss of extracellular matrix material, and loosely associated bacterial cells. These findings collectively demonstrate the effective inhibitory action of FB1-4 CFS on MRSA biofilm formation (Fig. 2C through E).

As illustrated in Fig. 2F, FB1-4 CFS suppressed EPS production by MRSA in a concentration-dependent manner. At sub-inhibitory levels (1/4× MIC and 1/2× MIC), FB1-4 CFS showed a markedly greater ability to reduce EPS synthesis relative to vancomycin at the same concentrations. Conversely, under higher treatment intensities (2× MIC and 4× MIC), vancomycin led to more pronounced EPS inhibition. Together, these findings indicate that FB1-4 CFS possesses a more stable anti-MRSA profile, particularly by maintaining effective suppression at sub-inhibitory concentrations where vancomycin is less effective.

### Analysis of antimicrobial components

**Fig 3:**
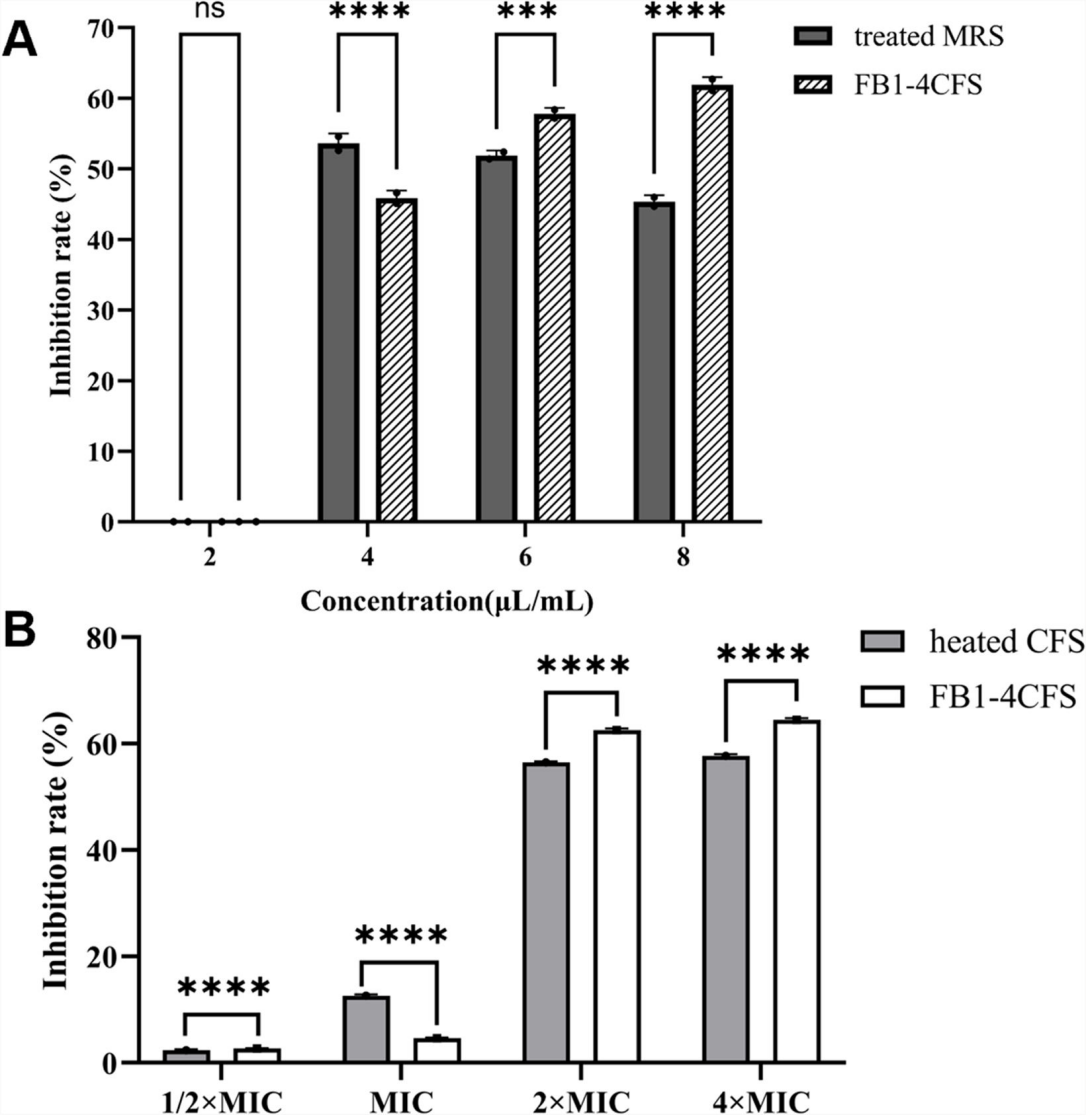
Evaluation of the antimicrobial components in FB1-4 CFS. (**A**) Effect of pH on antibacterial activity. The inhibitory rates of MRSA treated with FB1-4 CFS at MIC and pH-adjusted MRS medium (acidic control) were compared over time. (**B**) Effect of heat inactivation on antibacterial activity. The inhibitory rates of MRSA treated with native or heat-inactivated (121°C, 15 min) FB1-4 CFS at various multiples of the MIC were assessed. Data are presented as mean ± SD (*n* = 3). Statistical significance is indicated by (*P* < 0.0001) compared to the control. ns (not significant), ***P < 0.001；****P < 0.0001.

To investigate the material basis of the antibacterial activity of FB1-4 CFS, assessments were conducted from two perspectives: the contribution of pH and thermal stability. As shown in Fig. 3A, at the early stage of co-culture (4 h), the inhibition rate of MRS medium adjusted to the same pH as CFS (acidic control) against MRSA was 53.6%, which was significantly higher than that of FB1-4 CFS (45.9%, *P* < 0.0001), indicating that the acidic environment was the primary factor responsible for early inhibition. Upon extending the incubation to 8 h, the inhibition rate of the acidic control decreased to 45.4%, while that of FB1-4 CFS increased to 61.9% (*P* < 0.0001), suggesting that in addition to acidic substances, FB1-4 CFS contains other antibacterial components that exert more sustained effects at later stages.

Results from the thermal stability assay (Fig. 3B) further demonstrated that the core antibacterial components in FB1-4 CFS exhibit good heat resistance. At concentrations of 2× MIC and 4× MIC, the inhibition rates remained at 56.49% and 57.71%, respectively, after treatment at 121°C, with approximately 90% of the activity retained. However, at 1× MIC, the inhibition rate after heat treatment (12.63%) was significantly higher than that of the untreated group (4.6%), suggesting the possible presence of heat-labile substances in the native CFS that may exert a certain inhibitory effect on the antibacterial activity. In summary, the antibacterial activity of FB1-4 CFS is primarily attributable to heat-stable components, which provide a theoretical basis for its potential application in thermally processed food products.

### Whole-genome sequencing analysis of FB1-4

**Fig 4:**
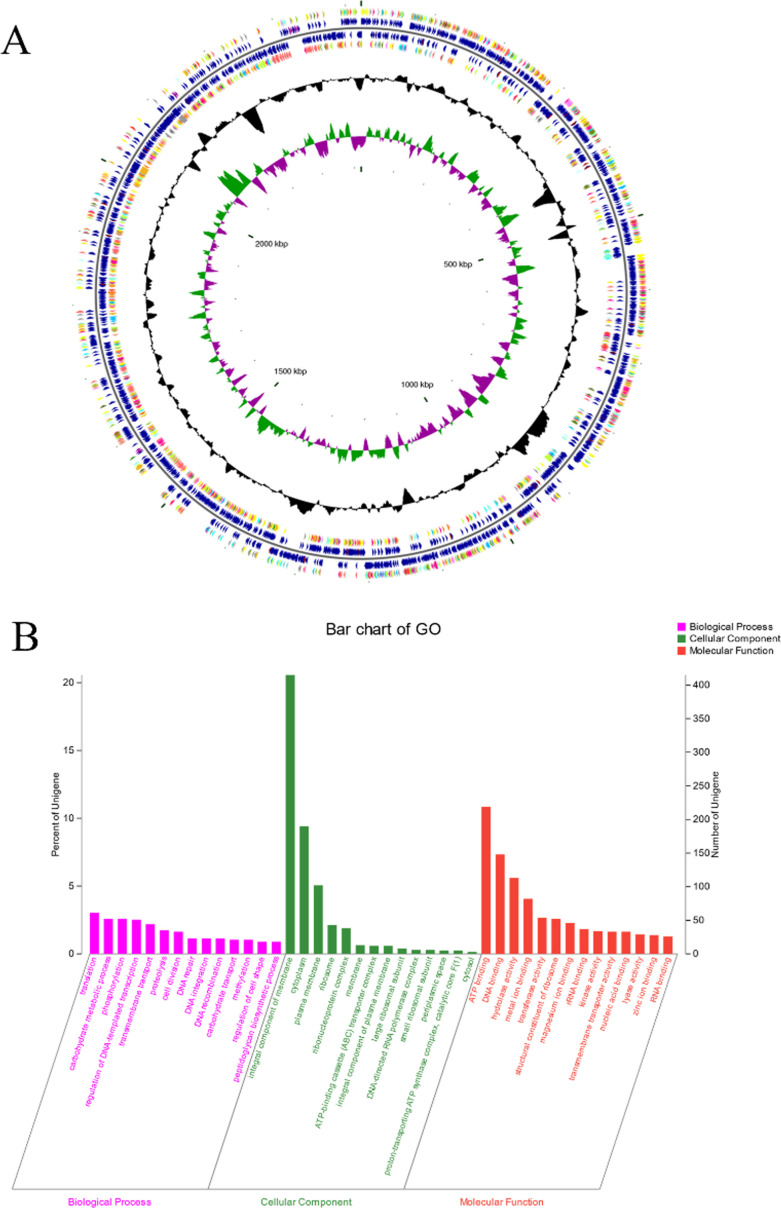
Genome circle map of *B. longum* FB1-4, generated using CGView. The genome circle map (**A**) comprises six concentric circles (outer to inner): (1) forward-strand CDS, color-coded by COG category; (2) forward-strand features (CDS, tRNA, rRNA); (3) reverse-strand features (CDS, tRNA, rRNA); (4) reverse-strand CDS, color-coded by COG category; (5) GC content, with outward/inward peaks showing regions above/below the genomic average; and (6) GC-skew, calculated as (G − C)/(G + C). (**B**) presents a visual analysis of GO term enrichment, categorized into three main groups: biological process, molecular function, and cellular component. The x-axis displays the most highly enriched GO terms within each category, while the y-axis shows the corresponding percentage of annotated genes.

FB1-4 possesses a circular chromosome of 2.42 Mb with a GC content of 60.24%, encoding 2,018 protein-CDSs, 56 tRNAs, and 16 rRNAs (Fig. 4A). GO annotation revealed notable enrichment of genes involved in translation (3.02%), carbohydrate metabolism (2.58%), phosphorylation (2.58%), and transmembrane transport (2.18%) (Fig. 4B). These genomic features suggest a high metabolic activity and potential for the synthesis and secretion of antimicrobial compounds, such as bacteriocins or metabolic effector molecules, supporting the observed antibacterial activity of its CFS.

### Analysis of transcriptome differences

**Fig 5:**
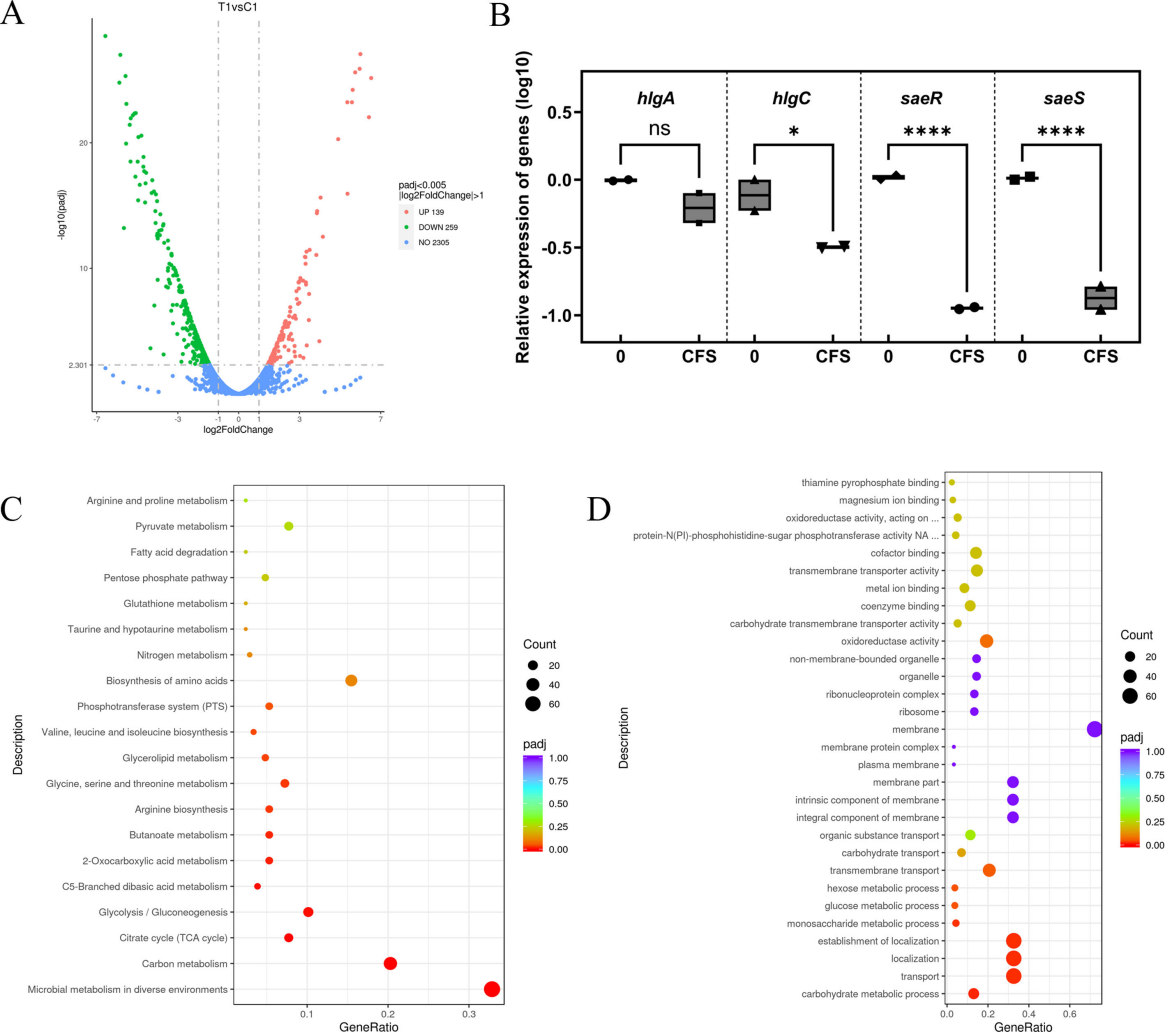
Analysis of differential gene expression and pathway enrichment at the transcriptomic level. (**A**) DEGs are visualized in a volcano plot. The log2 fold change is displayed on the x-axis, with the −log10 of the adjusted *P*-value on the y-axis. Significantly upregulated genes (UP 139) are shown as red dots, significantly downregulated genes (DOWN 259) as green dots, and genes without significant change (NO 2305) as blue dots. Significance thresholds are set at *P* < 0.005 and |log2FoldChange| > 1. (**B**) Expression levels of γ-hemolysin genes (hlgA and hlgC) and two-component system genes (saeR and saeS) were quantified by RT-qPCR. Data are presented as log10-transformed relative expression values. Box plots show the median, quartiles, and individual data points for each gene under control (0) and FB1-4 CFS treatment at sub-inhibitory concentration (1/2 MIC). Statistical significance between control and treatment groups is indicated as follows: ns (not significant), (*P < 0.05), and (****P < 0.0001). (**C**) Enrichment analysis of KEGG pathways for DEGs. The enriched KEGG pathways are shown on the y-axis, with the GeneRatio on the x-axis. The number of genes involved is indicated by dot size, and the adjusted *P*-value (*P*adj) is represented by color. (**D**) Enrichment analysis of GO terms for DEGs. The x-axis represents the GeneRatio, while enriched GO terms are listed on the y-axis. The dot size indicates gene count, and color denotes the adjusted *P*.

Transcriptome analysis was performed to investigate the molecular mechanisms underlying FB1-4 CFS-induced phenotypic changes. Comparison of gene expression profiles between FB1-4 CFS-treated (T1) and control (C1) groups, using stringent criteria (adjusted *P* < 0.005 and |log2FoldChange| > 1), identified 398 differentially expressed genes (DEGs) (Fig. 5A). Among significantly downregulated genes, key virulence genes related to antimicrobial mechanisms included the γ-hemolysin gene family (*hlgA*, *hlgC*) and two-component signal transduction system genes (*saeR*, *saeS*).

Treatment with FB1-4 CFS resulted in differential expression of virulence-related genes in MRSA. While the expression of *hlgA*, a member of the γ-hemolysin gene family, showed a non-significant decrease, *hlgC* expression was significantly downregulated by 47.8% (*P* < 0.05). The two-component regulatory system genes, *saeR* and *saeS*, exhibited significant downregulation of more than 80% (*P* < 0.05). These results indicate that FB1-4 CFS effectively suppresses multiple virulence factors in MRSA, including significant downregulation of the SaeR/S two-component system and its downstream target *hlgC* (Fig. 5B).

Following exposure to FB1-4 CFS, KEGG pathway analysis demonstrated substantial modifications in MRSA’s metabolic profiles. Among the significantly abundant pathways, microbial metabolism in diverse environments showed the highest representation (*n* = 68), followed by carbon metabolism (*n* = 42), while glycolysis/gluconeogenesis (*n* = 21) and TCA cycle (*n* = 16) also exhibited notable enrichment. The antimicrobial effects of FB1-4 CFS appear to be primarily mediated through its influence on energy-generating metabolic pathways. The analysis also uncovered significant disturbances in key amino acid synthesis pathways, encompassing the metabolism of glycine, serine, and threonine, along with the biosynthesis of valine, leucine, and isoleucine. These modifications suggest that FB1-4 CFS impairs MRSA’s cellular functions by disrupting protein synthesis and cell wall assembly, consequently suppressing bacterial growth and division (Fig. 5C).

The molecular basis of FB1-4 CFS antimicrobial action was further characterized through GO enrichment analysis of the DEGs. This investigation showed enrichment patterns across various biological processes, molecular functions, and cellular components. The most pronounced changes occurred within biological processes, where 24 genes were linked to carbohydrate metabolic processes. Additionally, three distinct yet related processes—transport, localization, and establishment of localization—each showed involvement of 60 genes. These observations indicate that FB1-4 CFS exhibits its bactericidal activity by targeting multiple metabolic pathways in MRSA, particularly affecting sugar metabolism, substance transport, and energy utilization systems, ultimately leading to metabolic dysfunction (Fig. 5D).

### Metabolomic profiling

**Fig 6:**
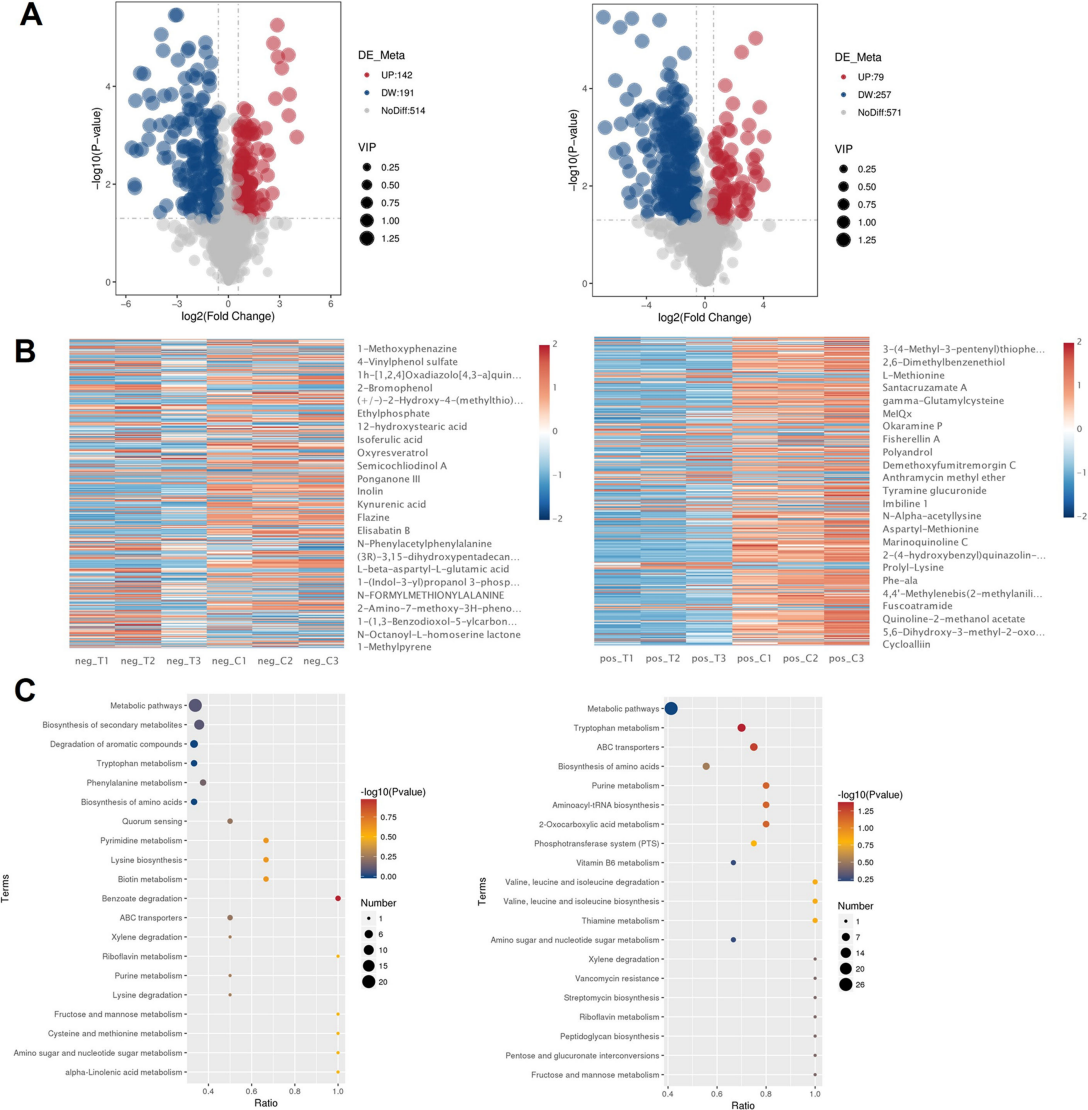
Differential metabolite analysis and pathway enrichment. (**A**) Volcano plots depicting metabolite changes in negative (left) and positive (right) ion modes. The y-axis shows statistical significance (−log10(*P*-value), while the x-axis represents log2(fold change). Red and blue dots indicate significantly upregulated and downregulated metabolites, respectively, with dot size proportional to VIP scores. Gray dots represent non-significant changes. (**B**) Hierarchical clustering heatmaps of differential metabolites in negative (left) and positive (right) ion modes. Dendrograms show sample clustering (vertical) and metabolite clustering (horizontal), with shorter branches indicating higher similarity. Color intensity represents the relative abundance of metabolites (red: increased; blue: decreased). (**C**) KEGG pathway enrichment analysis visualized as bubble plots for negative (left) and positive (right) ion modes. The x-axis shows the enrichment ratio (number of differential metabolites/total identified metabolites in the pathway). Bubble size indicates the number of differential metabolites in each pathway, while color intensity represents the significance level (−log10(*P*-value)) of the hypergeometric test, with darker colors indicating higher statistical significance.

Differential metabolite screening, employing stringent criteria (VIP > 1.0, |Fold Change| > 1.2, *P* < 0.05), identified 336 altered metabolites in ESI+ mode (257 decreased, 79 increased) and 333 in ESI− mode (191 decreased, 142 increased) (Fig. 6A). Two-dimensional hierarchical clustering analysis revealed distinct metabolic patterns between treated and control groups, with a predominant trend toward metabolite suppression in FB1-4 CFS-treated samples (Fig. 6B).

Metabolic pathway analysis through KEGG mapping revealed distinct alterations in multiple metabolic networks following FB1-4 CFS treatment. The most pronounced changes were observed in amino acid metabolism, particularly in lysine, tryptophan, and cysteine-methionine pathways. Several key metabolites exhibited significant downregulation (FC < 0.5, *P* < 0.01), including N-formylmethionine, L-lysine, kynurenic acid, 5-hydroxytryptophan, 3-indoleacetonitrile, phenylpyruvic acid, and (Z)-but-1-ene-1,2,4-tricarboxylate. Carbohydrate metabolism was also substantially impacted, with notable enrichment in fructose-mannose metabolism and amino/nucleotide sugar metabolism pathways, as evidenced by altered alpha-D(+)mannose 1-phosphate levels. Intriguingly, significant enrichment was detected in the quorum-sensing pathway, characterized by modulation of N-octanoyl-L-homoserine lactone and cis-2-dodecenoic acid, corroborating our *in vitro* observations of altered virulence factor expression and biofilm formation. Furthermore, the analysis unveiled significant perturbations in secondary metabolism networks, including aromatic compound metabolism, benzoate degradation, and xylene degradation pathways, demonstrated by systematic changes in aromatic metabolite profiles (Fig. 6C). These findings collectively indicate that FB1-4 CFS exerts comprehensive regulatory effects on MRSA metabolic networks, potentially targeting multiple cellular processes simultaneously.

### Effect of FB1-4 CFS on quorum-sensing system

**Fig 7:**
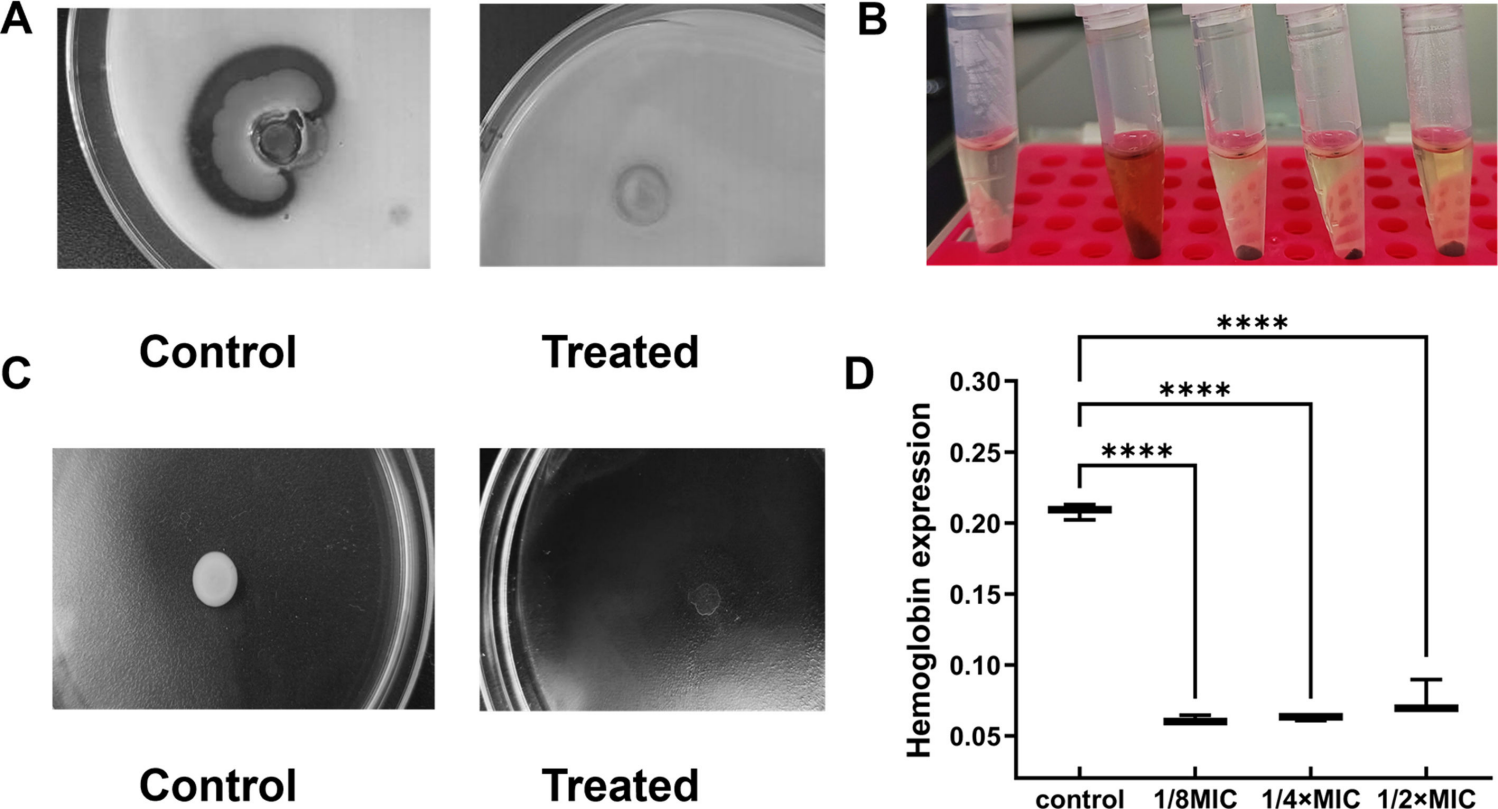
Effects of FB1-4 CFS on MRSA quorum-sensing-regulated virulence factors. (**A**) Protease activity assay showing clear zones on skim milk agar plates for untreated control (left) and FB1-4 CFS-treated MRSA (right) at sub-inhibitory concentration (1/2× MIC). (**B**) Hemolytic activity assessment using defibrinated rabbit blood. From left to right: sterile LB medium (negative control), untreated MRSA (positive control), and MRSA treated with increasing concentrations of FB1-4 CFS (1/8 MIC, 1/4 MIC, and 1/2× MIC). (**C**) Motility assay showing the spreading patterns of untreated (control) and FB1-4 CFS-treated MRSA (1/2× MIC) after 24-h incubation at 37°C. (**D**) Quantitative analysis of hemoglobin from rabbit red blood cells production under different FB1-4 CFS concentrations. Data are presented as mean ± SD (*n* = 3). Statistical significance is indicated by ****P < 0.0001 compared to the control.

The impact of FB1-4 CFS on MRSA quorum-sensing-regulated virulence factors was evaluated through multiple assays. Proteolytic activity assessment on skim milk agar demonstrated complete suppression of protease production in FB1-4 CFS-treated MRSA cultures, evidenced by the absence of proteolytic zones compared to untreated controls (Fig. 7A). In hemolysis experiments, MRSA cultures exposed to FB1-4 CFS at sub-inhibitory concentrations (1/8 MIC to 1/2× MIC) exhibited significantly reduced hemolytic activity. Quantitative hemoglobin analysis revealed that FB1-4 CFS treatment resulted in 65.6%–72.3% reduction in hemoglobin release (*P* < 0.0001) across all tested concentrations (Fig. 7B and D), indicating substantial inhibition of hemolysin secretion. Bacterial motility assays demonstrated that FB1-4 CFS treatment effectively restricted spreading of MRSA colony, as treated cultures remained confined to the inoculation site, while untreated controls showed extensive radial diffusion across the motility medium (Fig. 7C). These findings collectively demonstrate that FB1-4 CFS significantly attenuates multiple MRSA virulence mechanisms through the suppression of quorum-sensing-regulated processes, including protease production, hemolysin secretion, and cellular motility.

### *In vivo* mice experiment

**Fig 8:**
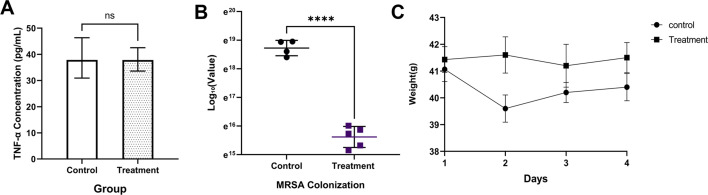
FB1-4 CFS intervention effects on MRSA colonization and host response in a mouse model. (**A**) TNF-α concentrations in control and treatment groups. No significant difference was observed (ns). (**B**) MRSA colonization levels (CFU/g) in control and treatment groups. Treatment significantly reduced MRSA colonization (****P < 0.0001). (**C**) Average weight of mice over 4 days in the control and treatment groups. Error bars represent the standard deviation from experiments.

The results of the study revealed important insights into the inflammatory response of MRSA-infected mice. Specifically, the TNF-α levels in the serum of MRSA-infected mice (control group) and the intervention group (treatment group) were measured using the ELISA method, which showed a strong linear relationship in the standard curve with a correlation coefficient (*R*²) close to 1. The average TNF-α concentration in the control group was measured at 38.78 ± 7.05 pg/mL. In comparison, the treatment group exhibited a slightly lower mean concentration of 37.65 ± 4.93 pg/mL. Although the TNF-α levels in the treatment group were marginally reduced compared to the control group, this difference did not reach statistical significance (*P* > 0.05). Notably, the control group displayed a wider range of TNF-α levels, with some individual mice showing significantly elevated concentrations, indicating variability in the inflammatory responses among the infected subjects. Conversely, the treatment group demonstrated more consistent TNF-α levels, suggesting that the intervention may have contributed to stabilizing the inflammatory response (Fig. 8A).

In addition to the inflammatory markers, the study also assessed the colonization levels of MRSA in the mice. The average MRSA colonization level in the control group was found to be 1.39 × 10⁸ ± 4.02 × 10⁷ CFU/g, indicating a high level of bacterial persistence. In stark contrast, the intervention group showed a significantly lower colonization level of 6.38 × 10⁶ ± 2.19 × 10⁶ CFU/g. This substantial reduction in MRSA colonization, amounting to a remarkable 95.41% decrease due to intervention with FB1-4 CFS, underscores the effectiveness of this intervention in reducing MRSA persistence within the mouse colon (Fig. 8B).

Finally, the impact of MRSA infection on the body weight of the mice was closely monitored throughout the observation period. The control group experienced significant weight loss, particularly evident from day 1 to day 2, reflecting the adverse physiological effects of the infection. Although there was a slight recovery in weight observed on day 4, the overall weight remained below the initial level, indicating ongoing negative health impacts. In contrast, the intervention group exhibited a more favorable trend, with a gradual increase in body weight from day 1 to day 3, followed by a slight decline that kept their weight above the initial level. This comparison suggests that the FB1-4 CFS intervention may play a beneficial role in supporting body weight maintenance and improving the overall physiological condition of the mice following MRSA infection (Fig. 8C).

## DISCUSSION

This study systematically investigated the antibacterial efficacy and mechanism of the CFS from strain FB1-4 against MRSA through a series of controlled experiments. The results demonstrated that FB1-4 CFS exhibited significant anti-MRSA activity *in vitro*, with an average inhibition zone diameter of 17.70–18.20 mm and an MIC90 value of 5.56 mg/mL. At 2× MIC, FB1-4 CFS maintained a stable bactericidal rate exceeding 80% over 8 h, showing more sustained killing efficacy compared to vancomycin at an equivalent concentration. In food matrix experiments, FB1-4 CFS achieved a 99.99% reduction in MRSA viability within 8 h, indicating its potential application in the food industry.

Further mechanistic studies revealed that FB1-4 CFS could reduce antibiotic resistance in MRSA by decreasing its antibiotic tolerance and inhibiting biofilm formation. FB1-4 CFS enhanced the susceptibility of MRSA to methicillin in a dose-dependent manner, consistent with the mechanism of probiotic supernatants modulating resistance genes proposed by Abbasi et al. (36). Moreover, at sub-inhibitory concentrations, CFS inhibited the expression of biofilm-associated genes *Psmα* and *Ica* by 62.4% and 60.7%, respectively, exceeding the activity of metabolites produced by *Lactobacillus acidophilus* (37) and *Lactobacillus paracasei* subsp. *A20* (38), highlighting the advantage of FB1-4 CFS for targeted intervention at sub-MIC levels.

Integrated multi-omics analysis systematically elucidated the multi-target antibacterial molecular mechanism of FB1-4. Genomic studies revealed that FB1-4 may directly participate in the biosynthesis and transmembrane secretion of antimicrobial substances to exert its antibacterial effects. Transcriptomic analysis further confirmed that FB1-4 specifically suppresses the expression of the SaeR/S two-component regulatory system and its downstream quorum-sensing target gene *hlgC* in MRSA, thereby effectively disrupting the cascade expression network of virulence factors. Correspondingly, metabolomic investigations uncovered a multi-faceted interference mechanism of FB1-4 on key metabolic pathways in MRSA: by significantly affecting the content of N-formylmethionine in the lysine metabolism pathway, inhibiting the biosynthesis of α-D-mannose-1-phosphate in the central carbon metabolism network, and interfering with the production of the quorum-sensing signaling molecule N-octanoyl-L-homoserine lactone, FB1-4 induces systemic disruption of the MRSA metabolic network. These integrated multi-omics results clearly demonstrate that FB1-4 achieves potent inhibition of MRSA through synergistic targeting of transcriptional regulation, signal transduction, and metabolic networks.

Furthermore, FB1-4 interferes with the MRSA quorum-sensing system via a triple mechanism: complete inhibition of protease secretion, blockade of hemolysin release (reflected by a 72.3% reduction in hemoglobin), and restriction of bacterial motility and spread. This multi-target synergistic mechanism can circumvent the potential resistance risk associated with single-target therapies, providing a theoretical foundation for developing novel anti-MRSA strategies.

The *in vivo* therapeutic effect of FB1-4 CFS was validated in a mouse model. CFS treatment reduced MRSA colonization in the mouse colon by 95.41% (*P* < 0.0001), consistent with the probiotic-mediated colonization resistance reported by Kim et al. (39), indicating that FB1-4 CFS can exert a protective role in complex biological environments by reducing bacterial load.

In summary, this study clarifies the molecular mechanism by which FB1-4 inhibits MRSA growth, revealing its multi-target synergistic action in disrupting MRSA drug resistance, virulence expression, and metabolic networks. These findings provide a scientific basis for developing new strategies against MRSA infections. Future work should focus on purifying and functionally validating the active components, applying genetic tools to elucidate the role of the saeR/S-hlgC pathway in virulence suppression, and advancing pilot-scale fermentation and preclinical safety assessments to facilitate industrial translation.

## Data Availability

The genomic data from this study have been deposited in the National Center for Biotechnology Information (NCBI) RefSeq database under BioProject accession number PRJNA1332689. The RNA-seq data generated in this study have been deposited in the Dryad Digital Repository under the accession DOI https://doi.org/10.5061/dryad.8931zcs53. The raw non-targeted metabolomics data have been submitted to the Metabolights database under the project identifier MTBLS13481.
